# The Impact of Alcohol on L1 versus L2

**DOI:** 10.1177/0023830920953169

**Published:** 2020-08-28

**Authors:** Tom F. Offrede, Jidde Jacobi, Teja Rebernik, Lisanne de Jong, Stefanie Keulen, Pauline Veenstra, Aude Noiray, Martijn Wieling

**Affiliations:** University of Groningen, Netherlands; University of Groningen, Netherlands; Macquarie University; University of Groningen, Netherlands; University of Groningen, Netherlands; Vrije Universiteit Brussel, Belgium; University of Groningen, Netherlands; Haskins Laboratories; University of Potsdam, Germany; University of Groningen, Netherlands; Haskins Laboratories

**Keywords:** Acute alcohol consumption, articulation, speech, bilingualism

## Abstract

Alcohol intoxication is known to affect many aspects of human behavior and cognition; one of such affected systems is articulation during speech production. Although much research has revealed that alcohol negatively impacts pronunciation in a first language (L1), there is only initial evidence suggesting a potential beneficial effect of inebriation on articulation in a non-native language (L2). The aim of this study was thus to compare the effect of alcohol consumption on pronunciation in an L1 and an L2. Participants who had ingested different amounts of alcohol provided speech samples in their L1 (Dutch) and L2 (English), and native speakers of each language subsequently rated the pronunciation of these samples on their intelligibility (for the L1) and accent nativelikeness (for the L2). These data were analyzed with generalized additive mixed modeling. Participants’ blood alcohol concentration indeed negatively affected pronunciation in L1, but it produced no significant effect on the L2 accent ratings. The expected negative impact of alcohol on L1 articulation can be explained by reduction in fine motor control. We present two hypotheses to account for the absence of any effects of intoxication on L2 pronunciation: (1) there may be a reduction in L1 interference on L2 speech due to decreased motor control or (2) alcohol may produce a differential effect on each of the two linguistic subsystems.

## 1 Introduction

Much empirical research has been carried out on the effects of alcohol consumption on human behavior and cognition. Alcohol intoxication, for instance, impairs cognitive functions like planning and spatial recognition (e.g., Weissenborn & Duka, 2003) and reduces inhibitory control capacity of behavior as well as of attention (e.g., De Wit et al., 2000; Fillmore, 2007), which in turn has a decreasing effect on emotional responses such as fear (Curtin et al., 2001). It also often leads individuals to become more aggressive (e.g., Gustafson, 1999; Hoaken et al., 1998). At low doses, alcohol can improve psychomotor skills, probably due to its effects on anxiety reduction (Chin & Pisoni, 1997); however, at slightly higher doses, intoxication affects muscle coordination, harming fine motor control and rendering it slower and less accurate (Chin & Pisoni, 1997; Hollingworth, 1923, for tapping speed and hand stability; Marczinski et al., 2012, for fine finger dexterity).

Given this evidence, it should not come as a surprise that alcohol consumption also has an influence on linguistic processes. Alexandrov et al. (1998), for instance, investigated the effect of alcohol on semantic processing in bilinguals. In an EEG event-related potential (ERP) study, they demonstrated that bilinguals’ first (L1) and second (L2) languages are processed partially differently when they are under the influence of alcohol, with L2 affected more than L1 under intoxication conditions. Moreover, from a more interactional perspective, Smith et al. (1975)^1^ studied unstructured interactions between friends while sober and under the influence of alcohol. They noted that, at low levels of intoxication, there was an increase in the volume of communication, conversation initiations, and speech overlap, and participants acknowledged their interlocutor’s statements significantly less. At a higher dose of alcohol, there was even more overlap, but the volume of communication was levelled off or even reduced. The authors attributed these changes to the disinhibition effect of alcohol on emotional responses and, possibly, to deficits in cognitive processing.

The impact of alcohol on spoken language production has also been studied in terms of speech articulation (or pronunciation). Indeed, pronunciation is often a fair indicator of an individual’s inebriation state. Hollien et al. (2009) have demonstrated that even lay listeners (as opposed to clinical professionals) are able to assess the presence of intoxication in people’s speech. These listeners can also systematically rank the speakers’ severity of intoxication, even though they are unable to accurately estimate the exact level of inebriation (similar results were obtained by Schiel, 2011). Further, techniques have been developed to automatically identify whether a recorded speech sample was produced under alcohol intoxication based, for instance, on prosodic features and acoustic properties of the phones produced (e.g., Biadsy et al., 2011; Bocklet et al., 2011). Such articulation changes that occur under the influence of alcohol are often related to the aforementioned deficit in fine motor control. Studies that compared the speech of participants both under states of sobriety and intoxication reveal that, when inebriated, speakers have a lower speech rate, higher utterance duration, and more irregular speech rhythm; they produce more disfluencies; they lengthen their vowels and (partially) delete their consonants; and they have less precise control of their vocal cord vibration, which increases their pitch level variability (e.g., Barfüßer & Schiel, 2010; Baumeister & Schiel, 2010; Hollien et al., 2001; Pisoni & Martin, 1989; Schiel et al., 2010; Watanabe et al., 1994).

Although most of the evidence discussed thus far concerns the influence of alcohol on pronunciation in a native language, a few studies have also focused on non-native speech. Guiora et al. (1972) gave participants drinks with varying amounts of alcohol (from zero to 88 ml of liquor) and tested them in an aural-oral task, in which they had to repeat sounds from an unknown foreign language. The authors found that, at low levels of alcohol ingestion, the participants were slightly better at foreign language pronunciation than control subjects. After a certain dosage, however, articulation declined considerably. In another study, Tisljár-Szabó et al. (2014) asked Hungarian speakers to repeat tongue-twisters, some of which were words from a foreign language unknown to the participants. For most tongue-twisters, speakers made many more speech errors in the alcohol-influenced condition than in a sober state. However, the articulation of foreign words was apparently unaffected by intoxication. One concern with these two studies is that they did not measure performance in a known L2 per se; rather, they tested articulation in unknown languages. Renner et al. (2018) addressed this issue by testing the performance of German–Dutch bilinguals. These participants were either sober, having drunk water, or intoxicated, having consumed vodka and reached a blood alcohol level of around 0.4%. They had to speak freely in their L2 Dutch for two minutes about a given topic, and two Dutch native speakers blind to the experimental manipulation rated the bilinguals’ proficiency. The participants who had consumed alcohol had their language performance rated significantly better than those who had drunk water. Interestingly, this difference was accounted for by their pronunciation ratings; ratings for grammar, vocabulary, and argumentation were not different per group. Renner et al. (2018) attribute this result to alcohol’s disinhibiting effect, which would enable bilinguals to speak more fluently in their L2. They speculate that this might happen because alcohol reduces language anxiety; however, they did not measure their participants’ anxiety level, so this hypothesis remained untested. Importantly, in their research, the level of alcohol intoxication was very low, so they did not assess changes in language production caused by higher levels of inebriation.

The present study—which is an extension of Wieling et al. (2019)—was designed to further explore the apparently diverging effect of alcohol intoxication on L1 and L2 pronunciation. We asked Dutch and English native speakers to rate the L1 (Dutch) and L2 (English) pronunciation of individuals who were under varying degrees of alcohol influence. Following the aforementioned previous findings, we hypothesized that the speakers’ L1 pronunciation would receive lower (intelligibility) ratings, the higher the amount of alcohol consumed, and that their L2 accent would receive *higher* (nativelikeness) ratings with increasing inebriation levels, at least at low doses of alcohol (cf. Guiora et al., 1972). The constructs we use to measure pronunciation in L1 and L2—namely intelligibility and nativelikeness, respectively—are arguably not directly comparable. However, Jułkowska and Cebrian (2015) provide evidence that there is at least a moderate correlation between the degrees of intelligibility and nativelikeness of non-native English speech samples, as rated by native speakers of English. Hence, we will compare the intelligibility of L1 speech and nativelikeness of L2 pronunciation, all while keeping in mind the caveat that the two concepts are not identical.

## 2 Method

Speech samples were collected at *Lowlands Science*, a science outreach event at the three-day music festival *Lowlands*, in the Netherlands, in August 2018. L1 pronunciation ratings and L2 accent ratings were obtained at two different moments: the former were collected at the same festival, whereas the latter were collected through an online questionnaire in 2019, as described below. The study was approved by the Faculty of Arts Research Ethics Review Committee of the University of Groningen (approval number 57794491).

### 2.1 Participants

Pronunciation ratings were obtained for a total of 137 adult speakers, but only the samples of 80 individuals who fitted the criteria described below were included in this analysis. The speakers included in the analysis were native speakers of Dutch who spoke English as an L2 (45 females and 35 males), ranging in age from 20 to 64 years (*M* = 31, *SD* = 9.5). They were native speakers of Dutch and had no other L1s; all of them had been born and lived in the Netherlands when the data were collected. They rated their own English-speaking proficiency between 3 and 9 on a scale from 1 to 10 (*M* = 7.5, *SD* = 1.2). Blood alcohol concentration (BAC) levels ranged from 0 to 1.59. However, because there were no participants with BAC levels between 0.8 and 0.97 (a gap of considerable size), and only a total of 7 speakers with a BAC of over 0.8 (out of which 2 speakers only had a single rating), we excluded all speakers with BAC higher than 0.8 from the analysis. The mean BAC of the speakers included in the analysis was 0.14 (*SD* = 0.2). Other participants excluded from the analysis were those who had any sort of hearing, reading, or speech impairments, who declared to have consumed any type of drugs (besides alcohol) previously that day, and whose parents were not both native speakers of Dutch.

#### 2.1.1 L1 pronunciation ratings

The raters of the pronunciation in the L1 samples were native speakers of Dutch who fit all the aforementioned criteria, in addition to having a BAC level of 0. In total, the ratings provided by 106 individuals were included (66 females and 40 males). Their mean age was 30 years (*SD* = 8.4).

#### 2.1.2 L2 accent ratings

The raters of the L2 accents were 115 native US-born and -raised adult speakers of English without hearing problems (29 females, 84 males, 1 identifying as belonging to other genders, and 1 who did not wish to provide their gender information). They were aged between 20 and 81 years (*M* = 47.9; *SD* = 16) and came from various US states. These raters had different language experiences: some had never lived abroad while others had lived in various countries. Importantly, we found that, whether those speakers had lived in Europe, in other non-European countries, or had never lived abroad, there was no significant difference between their ratings.

### 2.2 Materials

The target L1 sentence that the speakers produced and the raters evaluated was, *Het was voorjaar en de zon scheen, iepen waren in bloei, water liep uit fonteinen, roeken vlogen rond en goudvissen, zo groot als dolfijnen schoten door het glinsterende water.* (Translation: “It was spring and the sun was shining, elms were in bloom, water came from fountains, rooks flew around and goldfish as big as dolphins were shooting through the glistening water.”) The L2 speech samples consisted of the first two sentences of the elicitation paragraph used in the Speech Accent Archive (Weinberger & Kunath, 2011): *Please call Stella. Ask her to bring these things from the store: six spoons of fresh snow peas, five thick slabs of blue cheese, and maybe a snack for her brother Bob.*

It could be argued that accent ratings of samples obtained from read texts may not be as reliable as judgments of spontaneous speech (Weinberger & Kunath, 2011). However, Munro and Derwing (1994) have provided evidence that nativelikeness ratings tend to be the same when the language sample concerned comes from a read text as when it is spontaneous speech.

An adapted version of the Foreign Language Classroom Anxiety Scale (FLCAS; Horwitz et al., 1986) was used to assess the speakers’ language anxiety. Seven of the 11 questions were used in the present study—those that focused more on speaking, rather than listening. They were translated into Dutch and adjusted to the context of the experiment; that is, references to language class were replaced with references to speaking English. The questions consisted of statements, such as “I start to panic when I have to speak English without preparation,” which were rated on a 5-point scale (1: strongly agree; 5: strongly disagree). Reliability of the 7-question scale was adequate (Cronbach’s alpha of 0.71), and a single measure of language anxiety was thus used by averaging the ratings to all questions (and inverting the scores of questions where higher ratings indicated less anxiety).

### 2.3 Apparatus

For the recording of the speech samples, instructions were presented on a 27-inch computer screen. The acoustic speech signal was recorded at 22.05 kHz with the AAA software package (Articulate Instruments Ltd) and a Shure WH20 XLR headset microphone. Ultrasound imaging data of the speakers’ tongue movements were also simultaneously collected, but these were not analyzed for the present article.

The L1 perception experiment was implemented in PsychoPy (version 1.90.3), and the speech samples were presented through a pair of Sennheiser HD 280 headphones. The L2 perception experiment, developed and executed through the LimeSurvey survey tool (LimeSurvey.org), took place on the participants’ own computers.

### 2.4 Procedure

#### 2.4.1 Speech production

The participants were first informed of the purpose of the experiment and signed an informed consent form. Then, they proceeded to fill in a questionnaire concerning personal background information (e.g., age, gender, education level, province of origin) and to answer the FLCAS. After this, the experimenter assessed their blood alcohol concentration using a certified professional breathalyzer.

Once this intake section was over, the participants proceeded to the testing booth, where they were seated approximately one meter away from the computer screen on which the instructions were displayed, and the microphone was positioned close to their mouth. The participants then read aloud several words and sentences (some of which are not analyzed here) and carried out a few diadochokinetic tasks (i.e., the quick repetition of a series of alternating sounds, which can involve the repetition of one syllable, such as “PA,” or several syllables, such as “PA-TA-KA”). When the participants were unable to complete the sentence during its recording, which happened in less than 5% of the cases, they re-recorded it. These re-recordings occurred predominantly for the diadochokinetic tasks (not reported in this article). The L1 and L2 target sentences were only recorded anew if the speaker took a long pause after pronouncing only a few words; for instance, because their flow of speech was disrupted or because they paused to ask questions. General disfluencies and accidentally skipped or mispronounced words were never the cause for re-recording the sentences used in this study.

#### 2.4.2 L1 pronunciation rating

The raters were presented with a subset of the previously recorded speech samples in a random order. They were asked to indicate how intelligible (i.e., “*duidelijk*”) the Dutch pronunciations were on a scale from 1 to 5 (1: very unintelligible; 5: very intelligible), and they were told that they did not need to listen to the entire sample to make a decision. The entire rating session was duration-limited to 2.5 minutes, so each individual rated a different number of samples (*M* = 6, *SD* = 2.7) depending on whether they listened to the entire audio samples. Some of these raters had also participated in the speech sample collection; in such cases, it was ensured that they were only presented with samples provided by other speakers for rating.

#### 2.4.3 L2 accent rating

The LimeSurvey questionnaire was advertised on the Language Log website (http://languagelog.ldc.upenn.edu). The first section of the survey concerned personal background questions. Then, the participants were instructed that they would hear samples from native as well as non-native speakers of English; although they were only presented with samples of non-native speakers, we led them to believe they would also hear native accents so that their ratings would not be biased. They were then told that they should rate the nativelikeness of at least 10 speech samples on a 5-point scale (1: very foreign sounding; 5: (indistinguishable from a) native English speaker). At no moment was it mentioned that some of the speakers were inebriated. Again, the raters were informed that they did not need to listen to the entire samples to decide on a rating. On average each rater rated a total of 17 samples (*SD* = 9.2). As raters rated a random selection of audio samples, there was a very limited overlap between the samples rated by pairs of speakers. Instead of calculating the average rating per audio sample, we conducted an analysis in which each of the individual ratings was included.

## 3 Results

**Figure 1.**
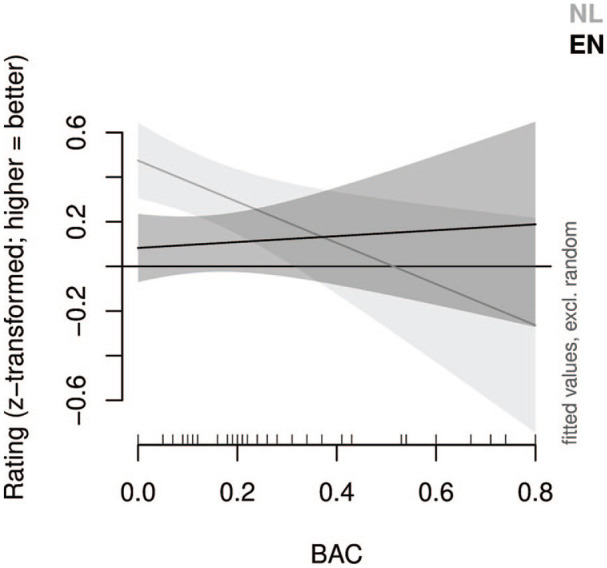
The generalized additive model of the relationship between blood alcohol concentration (BAC) and the ratings of the intelligibility (in L1, Dutch) and nativelikeness (in L2, English) of the participants’ speech. Although alcohol intoxication had a negative impact on L1 speech, this did not seem to be the case for L2 speech.

To assess the potentially non-linear influence of alcohol intoxication (measured in blood alcohol concentration, BAC) on the L1 and L2 ratings (*z*-transformed per speaker, but the results were similar when they were not transformed), we fitted a generalized additive model (cf. Wieling, 2018; Wood, 2017). To fit the model, we used the *mcgv* R package (R Core Team, 2019; Wood, 2011) and, to visualize the result, the *itsadug* package (van et al., 2017). We assessed the inclusion of random intercepts and slopes. No by-rater random intercept or slopes were significant; therefore, only a by-speaker random intercept and a by-speaker random slope for the produced language (i.e., English or Dutch) was included. The result of the model is visualized in Figure 1. The model showed a significant linear negative effect of BAC on the L1 (*p* < 0.01), whereas the somewhat positive (linear) effect of BAC on the L2 was not significant (*p* > 0.69). The difference between the slopes of the two lines was significant (*p* < 0.03). The residuals of the model followed a normal distribution.

In a subsequent exploratory analysis, we investigated whether other speaker-related variables significantly predicted the ratings. The resulting model is shown in Table 1. The higher the number of non-native languages spoken by the speaker, the higher the rating (for both L1 and L2). Additionally, a higher L2 language anxiety (FLCAS) and a lower self-rated English-speaking proficiency (which were also correlated at *r* = −0.7) are related to a lower rating for the L2. Importantly, the BAC effects remain unaffected. We also assessed whether BAC was related to foreign language anxiety. While the relationship was, as expected (Renner et al., 2018), somewhat negative, it did not reach significance (*p* = 0.21).

**Table 1.** Best-fitting exploratory model predicting ratings for L1 and L2.

Predictor	Estimate	Std. err.	*t*-value	*p*-value
(Intercept)	−0.804	0.618	−1.3	0.194
L1 (Dutch) vs. L2 (English)	1.510	0.894	1.689	0.091 .
Number of L2s spoken	0.145	0.045	3.21	0.001 **
BAC (L2)	0.041	0.264	0.156	0.876
BAC (L1)	−0.881	0.293	−3.006	0.003 **
Self-rated English proficiency (L2)	0.191	0.058	3.296	< 0.001 ***
Self-rated English proficiency (L1)	−0.006	0.066	−0.098	0.922
FLCAS (L2)	−0.329	0.115	−2.85	0.004 **
FLCAS (L1)	−0.162	0.134	−1.204	0.229

*** p < 0.001; ** p < 0.01; . p < 0.1.

## 4 Discussion

This study investigated the effect of alcohol intoxication on adult speakers’ pronunciation in a native as compared to a second language. We collected speech samples in the L1 and L2 of native Dutch speakers who spoke English as an L2 and who were under varying degrees of alcohol intoxication. We asked native speakers of Dutch and English to rate the pronunciation in those samples on how intelligible they were (for the L1) or how much they resembled a native accent (for the L2). Confirming our first hypothesis, higher BAC levels predicted poorer pronunciation in the participants’ L1. This is in line with numerous previous studies (e.g., Hollien et al., 2001; Pisoni & Martin, 1989; Tisljár-Szabó et al., 2014; Watanabe et al., 1994), and is easily explained by reduced fine motor control when under the influence of alcohol. In contrast, our second hypothesis was not supported: intoxication bore no significant positive relation with L2 pronunciation, even at low levels of alcoholization (in contrast to the findings of Renner et al., 2018 and Guiora et al., 1972), and alcohol did not predict significant changes in language anxiety levels, as Renner et al. (2018) hypothesized. These results, however, are in line with and expand on Tisljár-Szabó et al.’s (2014) investigation in three ways. Firstly, our study produced similar results to those of Tisljár-Szabó et al., namely that alcohol has no effect on pronunciation in a foreign language. Secondly, we extended this finding to a foreign language that is spoken by the participants (as opposed to an unknown language). Finally, this is the first evidence of this kind that compares Dutch (L1) and English (L2).

The selective effect of alcohol on articulation in each language is intriguing; here, we present two hypotheses regarding the reason for this discrepancy. The first concerns the interference of L1 knowledge in L2 production. It is widely established that, while speaking in their L2, speakers often make use of their L1 phonological repertoire and prosodic patterns, which gives way to non-native accents. It is thus possible that the decline in fine motor control caused by alcohol attenuates the transfer of L1 phonetic features into the speaker’s L2 speech. If this is the case, an increase in L2 nativelikeness ratings could be expected for higher BAC levels, as higher amounts of alcohol would hamper motor control more strongly and further reduce the effect of phonological transfer. Nonetheless, it is possible that, although our participants benefited from this reduced interference effect, they reached a ceiling level in their pronunciation performance due to a simple lack of English phonology knowledge.

The second possibility is related to the cognitive and neural organization of the L1 and L2 phonological systems. Bilinguals are mostly thought to have a somewhat separate phonological system for each language they know, even though they interact and have overlap (e.g., Haigh & Jared, 2007; Hambly et al., 2013; Spalek et al., 2014), and although this degree of separation is modulated by the age of acquisition of each language (Kang & Guion, 2006; Roelofs, 2003). In addition, alcohol is known to impact distinct cognitive and neural components differently, with some of those systems remaining unaffected by it (e.g., Bisby et al., 2007; Tracy & Bates, 1999). Therefore, it is possible that alcohol intake produces an effect on linguistic (or otherwise cognitive) subsystems more entailed in L1 processing differently than it does on those involved in L2 processing. This would be in accordance with Alexandrov et al.’s (1998) aforementioned study.

An additional interesting result yielded by our exploratory analysis was that the number of L2s spoken by the speaker positively predicted pronunciation ratings. This may suggest that multilingual speakers have a greater speech motor repertoire and dexterity. In other words, they may be more able to map different speech movements onto distinct phonetic targets, as compared to individuals who only speak two languages and might thus be more prone to applying L1 coordination patterns onto their L2 speech. Evidently, however, further research is necessary to explore the reasons behind the discrepant effects of alcohol on L1 and L2 pronunciation.

There are several possible explanations for the differences between our findings and those of Renner et al. (2018) and Guiora et al. (1972). It is possible that the discrepancy occurred because those two other studies treated alcohol consumption as a categorical variable, whereas we treated it as continuous. Another methodological issue of Renner et al. may have been that, since their proficiency raters were asked to evaluate many linguistic competences at once, they may have conflated other variables with pronunciation. In our study, on the other hand, we asked the raters to focus specifically on the speakers’ accents, and all our speech samples consisted of the same sentence; therefore, this type of attribution error was likely reduced. At the same time, these differences in the questions asked of Renner et al.’s and our participants limit the comparability of our findings. Further, it is possible that alcohol had a facilitatory effect on the social aspect of spontaneous conversation in Renner et al., while the current study may have observed a different effect because it engaged distinct cognitive resources—i.e., those involved in reading aloud. Finally, our participants and those of Renner et al. had arguably been exposed to very different amounts of L2 exposure throughout their lives. This may have meant that alcohol facilitated the access of Renner et al.’s bilinguals to their implicit L2 phonology knowledge, whereas our participants’ pronunciation reached the aforementioned ceiling level due to a lack of language exposure.

Our study also faces certain limitations. On the one hand, conducting most of the data collection at a music festival allowed us to treat alcohol intake as a continuous variable. On the other hand, participants may have been more tired than in other situations (e.g., at home, or during lab studies) or under the influence of substances they did not report. This, however, is likely not a grave problem, since carrying out the analysis on the data of all participants (including those who reported drug consumption) yielded similar results. It could also be the case, as pointed out by one reviewer, that our inebriated participants produced slower speech (e.g., Hollien et al., 2001), which can be related to confounds such as L2 proficiency and frequency of use. However, a regression model revealed that there were no significant differences between inebriated and sober participants in terms of those factors, which suggests that they are not likely to be confounds. Finally, as was stated in the Introduction, our study compared the intelligibility of L1 pronunciation with the nativelikeness of L2 speech. Although these constructs are moderately correlated (Jułkowska & Cebrian, 2015), they are not identical. The conclusions drawn from these data should thus be further refined in future studies.

## 5 Conclusion

Despite the limitations of this study and of the partial differences between our observations and those of previous research, the idea that alcohol intoxication has a differential effect in a first and a non-native language seems to be supported. Although pronunciation in an L1 suffers a deficit in intelligibility when the individual is under the influence of alcohol, this effect does not seem to extend to L2 articulation. Rather, pronunciation in an L2 seems to be modulated by other factors such as proficiency and number of foreign languages spoken. We argue that this L1-L2 discrepancy could potentially be accounted for in terms of reduced transfer of L1 motor patterns onto L2 pronunciation, or to a selective effect of alcohol on each of the two linguistic subsystems. Ideally, future research should attempt to investigate the mechanisms that underlie such divergence.

## Supplemental Material

code – Supplemental material for The Impact of Alcohol on L1 versus L2 Click here for additional data file. Supplemental material, code for The Impact of Alcohol on L1 versus L2 by Tom F. Offrede, Jidde Jacobi, Teja Rebernik, Lisanne de Jong, Stefanie Keulen, Pauline Veenstra, Aude Noiray and Martijn Wieling in Language and Speech

## Notes

1. Evidently, many studies that have examined the effects of alcohol consumption are not very recent. This is true, in part, because of ethical concerns which make it a delicate issue to bring participants to a high level of intoxication (cf. Paredes, 1989). This was one of the advantages of the present study: no alcohol was administered experimentally; the inebriated participants had ingested alcohol voluntarily prior to the collection of data.
